# Morphofunctional Traits and Pollination Mechanisms of *Coronilla emerus* L. Flowers (Fabaceae)

**DOI:** 10.1100/2012/381575

**Published:** 2012-04-30

**Authors:** Giovanna Aronne, Manuela Giovanetti, Veronica De Micco

**Affiliations:** Dipartimento di Arboricoltura, Botanica e Patologia Vegetale, Università degli Studi di Napoli Federico II, Via Università 100, 80055 Portici, Italy

## Abstract

It is accepted that the papilionaceous corolla of the Fabaceae evolved under the selective pressure of bee pollinators. Morphology and function of different parts of *Coronilla emerus* L. flowers were related to their role in the pollination mechanism. The corolla has a vexillum with red nectar lines, a keel hiding stamens and pistil, and two wing petals fasten to the keel with two notched folds. Pollinators land on the complex of keel and wings, trigger the protrusion of pollen and finally of the stigma from the keel tip. Data on pollen viability and stigma receptivity prove that flowers are proterandrous. The results of hand-pollination experiments confirmed that insects are fundamental to set seed. Interaction with pollinators allows not only the transport of pollen but also the rupture of the stigmatic cuticle, necessary to achieve both allogamy and autogamy. Field observations showed that Hymenoptera, Lepidoptera, and Diptera visited the flowers. Only some of the Hymenoptera landed on the flowers from the front and elicited pollination mechanisms. Most of the insects sucked the nectar from the back without any pollen transfer. Finally, morphological and functional characteristics of *C. emerus* flowers are discussed in terms of floral larceny and reduction in pollination efficiency.

## 1. Introduction

In the evolutionary pathways of the Angiosperms, flowers developed the sole function of sexual reproduction and corolla features were selected to attain the vexillary function: petals make the flowers visible among the green leaves and distinguishable from flowers of other species. From the animal perspective, flowers are protein (as pollen) and carbohydrate (as nectar) sources necessary for reproduction and individual survival. A list of insects can be attracted by flowers of a single plant species, but to distinguish between pollen vectors and nonpollinating visitors, the fine study of insect behaviour is highly advisable [1, 2]. 

Papilionaceous flowers, as well as other keel blossom, are reported to show adaptation for and against bees which are their pollinators [3]. In these flowers, pollen is hidden from rainfall and the foraging of the insects; moreover, pollen is placed in such positions on the bees' bodies that it is difficult for them to brush it off [4].

Plants belonging to the Fabaceae family have papilionaceous corolla with a standard petal (vexillum), two wing petals (alae), and two keel petals (carina). The keel petals enclose the staminal column of ten stamens and a single style. A nectary is generally found at the base of the corolla.

The pollination mechanisms of the Fabaceae species have been consistently classified into four different types: explosive, valvular, piston, and brush, and several evolutionary pathways are reported among them [5–10]. In the explosive mechanism the staminal column is held under pressure within the keel, and when the tension is released, the same column snaps forward against the standard petal causing all the pollen to be instantly released. This process is also known as tripping and it is generally accomplished when the keel is pressed down by a visiting insect. Nevertheless, a certain amount of tripping can occur without insect visits, due to heavy rain or high temperatures that weaken the turgidity of the restraining keel tissues. Once tripping has occurred, the staminal column does not return into the keel. In the valvular mechanism, the upper rim of the keel is unsealed; it opens along its total length when the keel is moved downwards by the pollinator and closes when the insect leaves the flower. In this case pollen can be released repeatedly to numerous visitors. In the piston mechanism, the keel tip moves under the pressure of the insect while anthers and stigma keep their place. Pollen is released from the anthers and pushed out through a hole in the keel tip. It can be dispersed with repeated visits. In the brush mechanism, as the pistil is longer than the stamens, the stigma extends beyond the anthers, avoiding self-pollination; the upper part of the style develops erect trichomes acting as a pollen brush [11]. When sufficient pressure is exerted on the standard and wing petals, the pistil protrudes from the keel tip: the stigma comes first into contact with the insect receiving (if present) external pollen and the style brushes the pollen on the visitor. When pressure is released, style and stigma return to their former position inside the keel.

*Coronilla emerus* L. is a shrub species belonging to the subfamily Faboideae, tribe Coronilleae. It is quite common on the margins and in the gaps of broad-leaved forests and in the Mediterranean maquis. It is generally absent in highly disturbed ecosystems or markedly dry environments. Galloni et al. [10, 12] compared pollen presentation, pollen-ovule ratio, self-compatibility, and spectrum of pollinators of this species with those of other Mediterranean legumes. They showed that insect visits are necessary for seed production and suggested that a positive relationship occurs between pollinator specificity and reproductive success.

The aim of this work was to study morphological and functional traits of flowers of *C. emerus* L. and to analyse their role in the pollination mechanism.

## 2. Materials and Methods

Field observations, field experiments, as well as flower and insect sampling were performed on plants in the Gussone Park at Portici, southern Naples, Italy (40° 49^′ ^N; 14° 20′ E). The dominant feature of the park is its *Quercus ilex* L. forest.

In the field we analysed insect behaviour, approaching and handling the flowers. We observed insects visiting flowers of *C. emerus* during three sunny days and recorded the type of visit they pay the flower (front side visit, that is, approaching the flower from the front of the corolla and inserting their ligula among the petals; back visit, that is, approaching the flower from the back of the corolla and inserting the ligula at the base of the vexillum claw). We recorded details of flower handling from a total of 75 individual visits. We were able to capture several of these insects (11 after the front approach and 5 after the back approach). The insects were carefully observed under a stereomicroscope (Olympus SZX9) in order to record presence and position of pollen grains on the bodies. Glycerine jelly stained with basic fuchsine was used to remove pollen from the insect's body [13]. Taxonomic identification was achieved using a light microscope (Olympus BX-60).

We studied *flower morphology*, the opening mechanism of the keel petals and the subsequent anther release analyzing numerous flowers at different phenological stages. We observed the flowers either in the field or in the laboratory under the stereomicroscope. We examined insect flower handling ability by means of direct observations in the field.

To clarify the role of morphological traits in the pollination mechanism, we studied timing of pollen viability, stigma receptivity, and breeding system. *Pollen viability* was tested by fluorochromatic reaction (FCR) with fluorescein diacetate [14]. We added a fresh solution of fluorescein diacetate in acetone at 2 mg/mL to 10% sucrose solution up to saturation. We deposited one 0.02 mL droplet on each pollen sample, mounted the slides with a cover slip, and kept them in the dark. Observations were made within 5 minutes with an Olympus BX-60 microscope equipped for reflected-light fluorescence with UV lamp, bandpass filter 330–385 nm, dichromatic mirror 400 nm and above, and barrier filter 420 nm and above. For each slide we recorded through a digital camera (Olympus Camedia C4040) not less than five field views, accounting for a minimum of 500 total pollen grains. Classification and counting of fluorescent pollen grains was obtained using the image analysis system Plant Meter [15]. The effect of temperature and relative humidity on pollen viability was measured on samples of pollen collected from just dehisced anthers of five plants. Pollen was spread into uniform layers on microscope slides and transferred into sealed Petri dishes. Different humidity conditions were obtained by lining Petri dishes with grains of CaCl_2_ (RH = 0) and with moist filter paper (RH = 100). Pollen was also exposed to 4°C, 20°C, and 37°C for 4, 7, and 22 hours. Results were organised into a factorial design (2 conditions of humidity, 3 levels of temperature, and 3 time intervals) and compared with an ANOVA by means of the SPSS statistical package (SPSS Inc., Chicago, IL, USA).

*Stigma receptivity* was tested on different samples of 20 flowers tagged at anthesis and processed daily until the very end of flowering. Receptivity was indirectly evaluated by assessing the esterase activity [16].

The *breeding system* was studied by performing hand-pollination experiments on a total number of 195 flowers equally distributed on three plants. To prevent insect visits, we covered 135 of these flowers with a fine muslin net and we left the remaining 60 to be free-pollinated. The first group was divided into three subsets of 45 flowers: (a) untouched (free-pollinated), (b) hand-pollinated when in full bloom using a bulked sample of pollen freshly collected from flowers of different plants (cross-pollinated), and (c) hand-pollinated when in full bloom using pollen freshly collected from several flowers on the same plants (self-pollination). We removed the nets three weeks after the complete end of flowering.

## 3. Results

### 3.1. Morphology and Floral Biometry

**Figure 1:**
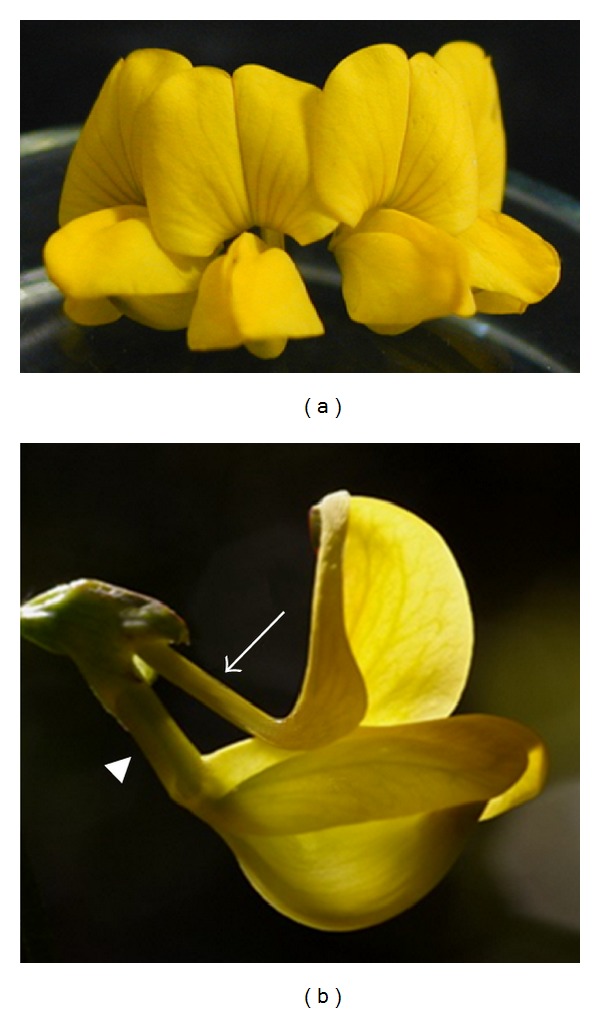
Flowers of *C. emerus*. (a) Front view of the flower with the wings-keel complex. Red nectar guides converging in the claw are evident on the standard petal. (b) Lateral view of the flower. The arrow indicates the standard claw. The arrowhead points to the cylindrical part of the keel.

**Figure 2:**
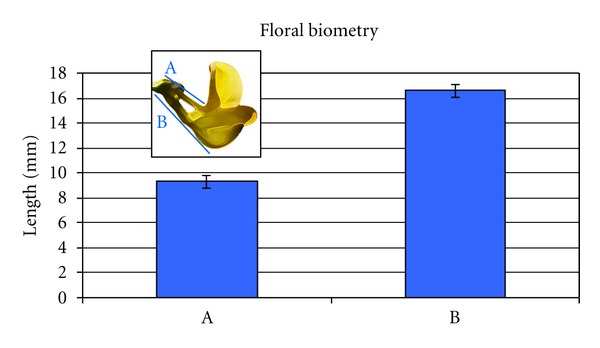
Floral biometry of *C. emerus*. Data show measurements as in the figure. Bars indicate standard deviation.

Flowers of *C. emerus* L. are born on long pedicels and form a crown-shaped inflorescence with 1–8 flowers, often 5. Single flowers are yellow and have a standard petal, two wings, and the remaining two bottom petals growing together to form the keel. Red lines are evident on the standard petal (Figure 1(a)). The lines converge in the claw which is thin and detached from other petals (Figure 1(b)). The keel has a proximal cylindrical part twice as long as the standard claw (Figure 2). Its distal part forms a pressed angular pouch, with an acute porate tip. 

**Figure 3:**
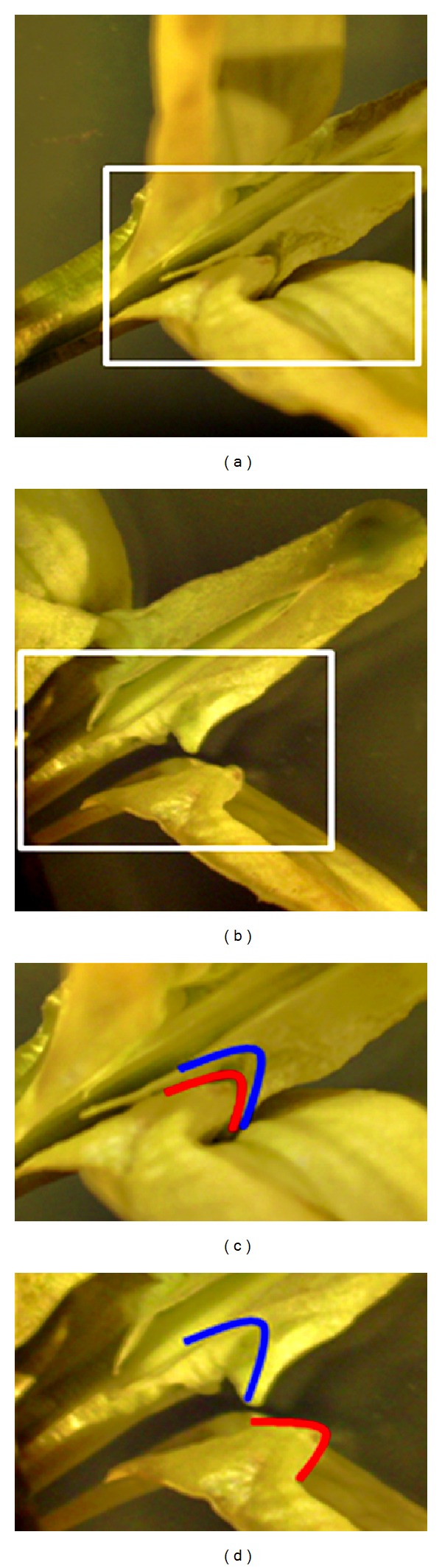
View from above of the wings-keel complex of *C. emerus* flowers. The notched folds are in the natural position in (a) and (c) while they are detached in (b) and (d).

The keel and the wings are attached by means of two notched folds (Figure 3). The complex of keel and wings serves as a landing platform for insects visiting the flower from the front.

**Figure 4:**
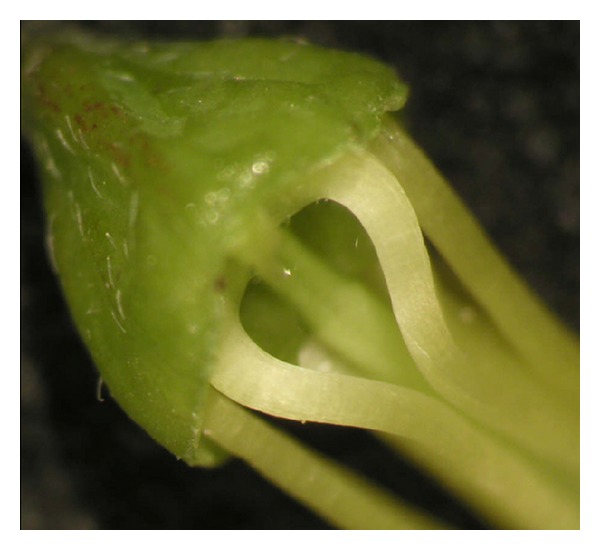
Flower base of *C. emerus* with the nectary. Two small fenestrae are evident between the joined and the free filaments.

The ten stamens are diadelphous. Nine filaments are fused by the basal part into a sheath open along the upper side. The tenth is free and lies on the others. A nectary is placed at the base of the flower limited to the area of the receptacle. Between the joined and the free filaments there are two small fenestrae which allow access to the nectar (Figure 4).

**Figure 5:**
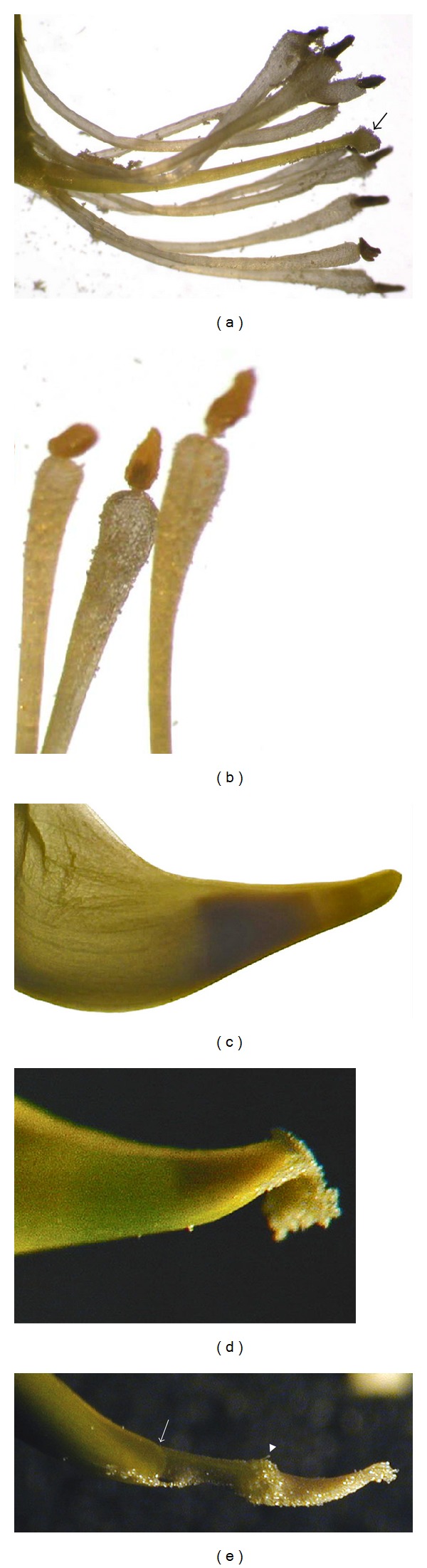
(a) The bunch of style and filaments of *C. emerus*; the arrow points out the capitate stigma. (b) Clavate and flat apex of filaments with the erect anthers. (c) The style and free parts of the filaments clamped into the keel tip. (d) Pollen squeezed from the keel tip. (e) Pollen, stigma (arrowhead), and upper part of the style protruding from the keel petals (arrow).

The distal parts of the filaments are free (Figure 5(a)). They are clavate and have an enlarged flat apex. An erect anther tops the filament (Figure 5(b)). The ovary lies in the sheath of the filaments along the cylindrical part of the keel (Figure 1(b)). It has a long style with a capitate stigma. The whole pistil has the same length as the stamens (Figure 5(a)). The style and the free parts of the filaments are clamped into the keel tip (Figure 5(c)). 

### 3.2. Pollination Mechanism

The staminal column is not held under pressure within the keel; therefore no tripping can occur during insect visits. If flowers are untouched, stamens and pistil are never exposed and remain enclosed in the petal keel. Insects landing on the front of the flower ride the wings-keel complex. Their legs push down the wing petals which pull back the keel by the notched folds. Repeated insect visits do not detach the wings from the keel. The back forward movements of the keel press firmly both anthers and stigma into the acute tip of the keel. After anther dehiscence, the clamped heads of the filaments prevent pollen moving back and the latter is forced to squeeze through the hole (Figures 5(d) and 5(e)). Subsequent visits release separate bulks of pollen. When sufficient pressure is exerted by a visiting insect, the stigma also protrudes from the keel petals but returns to its former position inside the keel when pressure is released. During the visit, pollen and stigma are pressed against the underside of the insect, priming the pollination process.

### 3.3. Functional Traits

**Figure 6:**
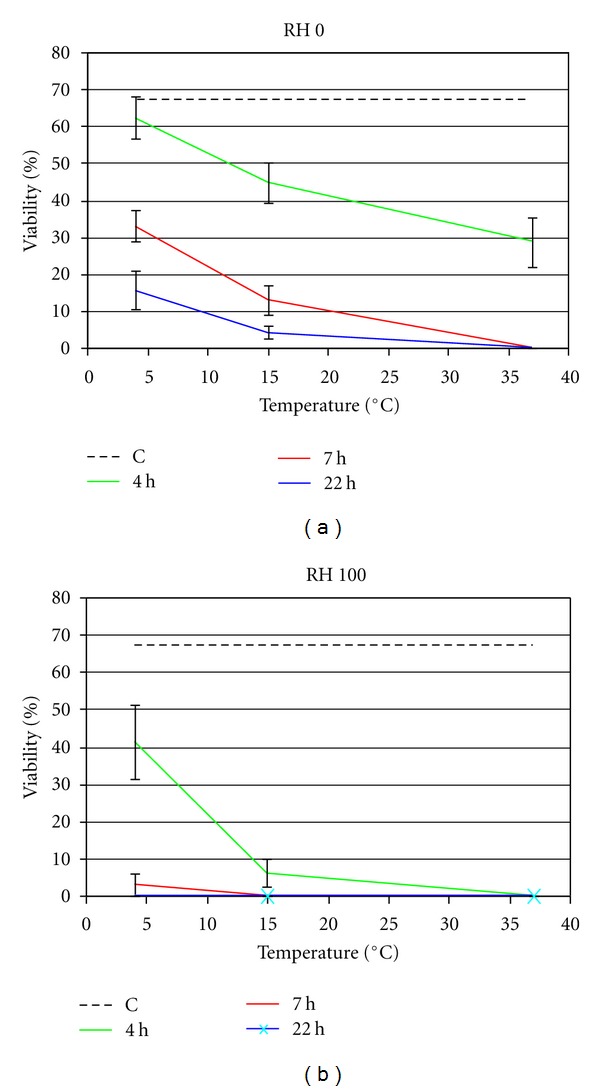
Effect of temperature (4, 15, and 37°C) and time (4, 7, and 22 hours) on pollen viability of *C. emerus* under dry (RH 0) and wet (RH 100) conditions. Dotted lines indicate viability at the beginning of the experiment and bars confidence intervals (*P* < 0.05).

Stigma became receptive at the end of anthesis which lasted on average three days. Positive reaction was obtained after scratching the stigmatic cuticle. Results of the experiment on the effects of relative humidity and temperature on pollen viability showed that viability at dehiscence was 67% (Figure 6). Under dry conditions, after 4 hours at 4°C, pollen viability was statistically not reduced, while it was significantly lower when pollen was left at higher temperature. Longer storage of the pollen, under the same conditions, showed a similar trend but with a further loss of viability at all temperatures. After 22 hours at 15°C and 37°C no viable pollen was found. At high relative humidity, after 4 hours at 4°C, a dramatic reduction in viability occurred while almost no viable pollen was found at 15°C and 37°C. Pollen stored for a longer period was not viable at all temperatures. The overall analysis of these data showed that pollen is viable at the beginning of anthesis while the stigma is not yet receptive. *C. emerus* is therefore a proterandrous species.

**Figure 7:**
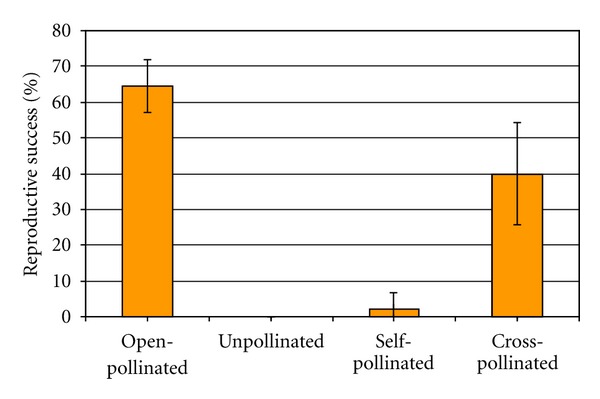
Hand pollination experiment in *C. emerus*. Reproductive success is expressed as percent of flowers which developed pods with seeds. Bars indicate confidence intervals (*P* < 0.05).

Investigation on the breeding system showed that 64% of the free-pollinated flowers developed pods with seeds (Figure 7). Within the hand-pollination experiments, cross-pollinated flowers achieved the best fruit-set. Only 2% of the self-pollinated flowers developed fruit while untouched flowers never set fruit.

### 3.4. Insect Visitors

**Figure 8:**
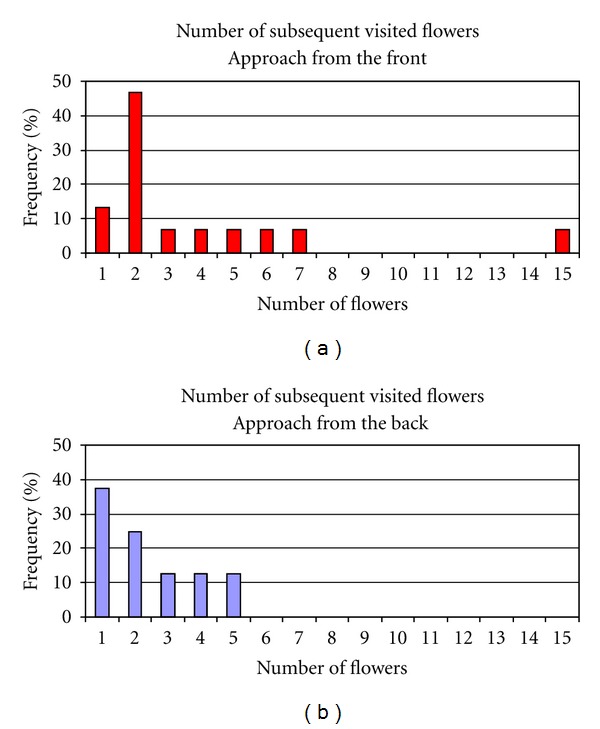
Number of *C. emerus* flowers subsequently visited by the same insect. (a) Insects approaching the flowers from the front. (b) Insects approaching the flowers from the back.

Regardless of their taxonomy, out of a total of 75 carefully observed insects, three quarters approached the flowers from the front and one quarter from the back. The insects visiting the flowers of *C. emerus* belonged to different taxa of the orders Lepidoptera, Diptera, and Hymenoptera (Table 1). Each insect taxon always approached the flower from the same side and maintained consistent behaviour during the visits. The individuals belonging to Lepidoptera and Diptera approached the flower from the back of the corolla and sucked nectar by inserting the glossa into the nectary at the base of the standard claw. The same approach was adopted by *Apis mellifera*, the only Hymenoptera not having contact with the carena and then with the pollen. By contrast, other Hymenoptera belonging to the genera *Anthophora, Eucera, Megachile, *and* Bombus* visited the flowers from the front side of the corolla, inserting the glossa under the standard petal to reach the nectar. The mean number of subsequent visits to *C. emerus* flowers did not statistically differ between insects approaching the flower from the front and those from the back. However, most of the insects approaching the flowers from the front visited two flowers consecutively, and in some cases they visited many more (up to 15) before leaving (Figure 8(a)). Insects approaching the flowers from the back used to visit fewer flowers and more often only one (Figure 8(b)).

Observations on flower handling showed that, while an insect was grasping the keel and sucking nectar, pollen was deposited on the ventral side of its body. However, we observed *Anthophora* sp. collecting not only nectar but also pollen. After sucking the nectar they used to move back on the wings-keel complex and to start rolling their front legs to remove the pollen. The small bee belonging to the Halictidae used to approach the flower from the front, land on the keel, and turn 180 degrees. As a result, the head was in front of the keel hole and the median and back legs pushed the wings-keel complex to get pollen which was promptly eaten. We never observed this type of bee collecting nectar.

**Figure 9:**
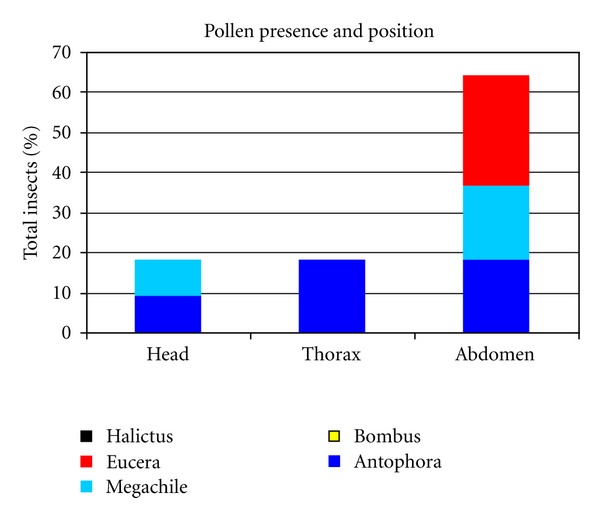
Presence and position of *C. emerus* pollen on different parts of the body of visiting insects. Data are expressed as percent of the total insects analysed.

Records of the presence and position of *C. emerus* pollen on the body of visiting insects confirmed those of flower handling (Figure 9). No pollen was found on the body of the Halictidae and *Bombus* individuals. The three remaining taxa showed pollen on the abdomen in about 60% of the analysed insects. Slightly less than 20% of the insects bore pollen on the head; they belonged either to *Megachile* sp. or to *Anthophora* sp. We found pollen on the thorax only of *Anthophora* sp. No pollen grain belonging to plant species other than *C. emerus* was found on any of the analyzed insects.

## 4. Discussion

Most bee species are believed to have evolved structural and behavioural adaptations to collect and transport pollen from a variety of flowers [17]. At the same time, it is widely accepted that flower morphology undergoes selective pressure by pollinators [18, 19]. The papilionaceous corolla is considered a general adaptation to pollination by Hymenoptera [3] and a closer view of the papilionaceous flowers of Fabaceae showed the occurrence of four different mechanisms of pollen release [5, 8]. The pollination mechanism of *C. emerus* is classified as piston type [10]. We showed that refined flower morphological traits have evolved in the flower of this species. As a consequence, pollen release and stigma protrusion occur only when the mechanism is properly elicited. The bundle of filaments with the enlarged flat apex prevents pollen spreading in the keel and forces all the pollen to be squeezed under the insect abdomen. In *C. emerus*, subsequent sternotribic pollination events can occur within a single flower. Therefore, all pollen produced within a flower can be potentially delivered to those insects able to trigger the mechanism without any waste.

Given that, in the piston mechanism of pollen release, a massive amount of self pollen lies on the stigma, we wanted to verify the occurrence of functional traits able to avoid self-fertilisation phenomena. It is generally assumed that in proterandrous flowers self-fertilisation is avoided because pollen is viable and released before the stigma becomes receptive. However, to avoid selfing, the earlier release of the pollen must be accompanied by a rapid loss of its viability [20]. This occurs in *C. emerus*: we showed that either all pollen grains or the great majority of them were not viable after one day at any combination of environmental factors.

In the majority of Angiosperms the water content of pollen grains changes during the period from formation to germination: pollen grains dehydrate before dispersal, are in equilibrium with the environment during dispersal and drain water from the stigma after landing [21]. We showed that in *C. emerus* the reduction rate of pollen viability is highly dependent on both temperature and humidity conditions: the warmer and more humid the environment is the quicker is the loss of viability. It is reported that pollen hydration activates physiological processes that make the pollen more susceptible to heat stress [22]. Moreover, tolerance of high and low temperatures and RH is variable among the species [23, 24]. As pollen of *C. emerus* is not exposed before dispersal, we assume that it does not undergo severe dehydration prior to pollination. That said, the limited lifespan of pollen grains of this species does not prevent reproductive success due to the rapid transport by pollinators.

The stigmatic cuticle is a further mechanical barrier to avoid self-fertilization. Its presence in *C. emerus* was reported by Galloni et al. [10], while its structural characteristics and role in other species of Fabaceae were previously described [25]. In our hand-pollination experiments, reproductive success was lower than in free-pollinated flowers even in the case of cross-pollination. It might be that the stigmatic cuticle was not properly ruptured during hand-pollination performance. Nevertheless, a small number of self-pollinated flowers developed fruits, confirming that *C. emerus* is not completely self-incompatible. Given that untouched flowers never developed any fruit, the visit of those insects able to trig the mechanism is fundamental to elicit not only allogamy but also autogamy.

In the papilionaceous corolla, the vexillum attracts the bees and the keel hides the sporophylls. This should prevent the collection of pollen solely for bee brood development, while pollen is placed on the bee body for its transfer to another flower. The specific function of the wings is generally neglected, but we showed that in *C. emerus* it is their morphology and correct handling that elicit pollen release and stigma protrusion.

The above considerations emphasize the evolution of several morphological and functional traits combined together to optimize pollination process in *C. emerus*. Indeed, the attraction of proper pollen vectors and the efficient location of the grains establish selective pressure, reducing the quantity of produced pollen. Nevertheless, our data showed that a considerable part of the insect visitors adopted an unsuitable approach to the flower: more than a fifth of the insects visited the flower from the back, without working as pollen vectors.

Insect belonging to Lepidoptera and Diptera mismatched floral morphological characteristics and did not elicit the pollination mechanism. During visits they collected only nectar while pollen remained safely hidden. Applying the floral larceny terminology [26], honey bees are also basic working visitors and act as nectar thieves on flowers of *C. emerus*. It is well known that, compared to other bee species, the honey bee collects an enormous quantity of nectar. The interest of this bee in flowers of *C. emerus* is confined to nectar collection probably because its body size is unsuitable to trigger the pollination mechanism.

The evolution and functional role of floral robbery is at present an intriguing and not completely clarified matter. It has been generally described as a phenomenon precluding the possibility of pollination, but in some cases it has been reported to improve floral set [27]. Further work should be conducted to discuss flower-insect interactions in terms of floral larceny and pollination efficiency of *C. emerus*.

In the light of our findings, we can make the following considerations. Among the insects approaching the flower from the front, those able to elicit the pollination mechanism belong to the long-tongued bees. Biometric data show that shapes and sizes of different floral parts match those of the proboscis and body of this functional group of insects [28, 29]. However we did not find pollen on *Bombus* species: they might have removed the grains from the body, thereby precluding the possibility of cross pollination. Pollen was found on the abdomen, thorax, and head of* Anthophora*, suggesting that the whole pollination system is less efficient than in *Eucera *and* Megachile.*

The small bees belonging to the Halictidae showed peculiar behaviour. They collected pollen from the keel tip but no grain was found on their bodies. They can be considered pollen thieves although their role as pollinator cannot be completely ruled out.

As a general conclusion, flowers of *C. emerus* need pollen vectors to produce seeds and legitimate pollinators receive only nectar as reward. Nevertheless, flowers also attract less efficient pollinators and visitors not involved in pollination. Removal of nectar by robbers may reduce pollination efficiency of legitimate insects: their subsequent visits to the same flower are shorter because there is less reward to be removed [30]. In these cases, the refined morphological and functional characteristic of the flower hides and precludes access to pollen and the receptive stigma. In the evolutionary perspective this can limit antagonist insects in favour of mutualism between flower and a few specific insects.

## Figures and Tables

**Table 1:** Insects observed while paying visits to *C. emerus* flowers, from the front of the corolla or from the back.

Approach from the front			Approach from the back		
Order	Family	Genus	Order	Family	Species
Hymenoptera	Anthophoridae	*Anthophora sp.*	Hymenoptera	Apidae	*Apis mellifera*
		*Eucera sp.*	Lepidoptera		
	Megachilidae	*Megachile sp.*	Diptera		
	Apidae	*Bombus sp.*
	Halictidae	
